# Pancreatitis from posterior gastric wall perforation by ingested metallic wire—case report and review of literature

**DOI:** 10.1259/bjrcr.20230070

**Published:** 2023-09-11

**Authors:** Gayatri Senapathy, Sudhakar Vengala, Rohini Muriki, Hardik Rughwani, Rakesh Kalapala

**Affiliations:** 1 Department of Radiology, Asian Institute of Gastroenterology, Gachibowli, Hyderabad, India; 2 Department of Gastroenterology, Asian Institute of Gastroenterology, Gachibowli, Hyderabad, India

## Abstract

Gastric and duodenal perforation from ingested organic and inorganic foreign bodies, such as sewing needles, toothpick, metallic wires, fish and chicken bone, are uncommon incidents as most foreign bodies pass in the faeces. The perforated foreign body can at times migrate and either penetrate causing traumatic injury or incite inflammation with formation of abscesses or pseudo-tumours in the adjacent organs such as the liver and pancreas. We report one such case of pancreatitis in a child resulting from a metallic wire perforating the posterior gastric wall and penetrating the pancreas. The findings were detected on CT and the foreign body was extracted endoscopically. We also present review of literature on similar case reports.

## Case report

A 10-year-old boy had complaints of pain abdomen in the epigastric region radiating to back associated with multiple episodes of vomiting in the last 3 days. Initial ultrasonography of the abdomen done elsewhere at a clinic was normal at presentation. In view of progressively increasing upper abdominal pain and vomiting, the child was brought to our institute, a tertiary care centre, for further work-up.

A repeat ultrasonography was performed on Day 4 and revealed mildly bulky pancreas, fluid in the lesser sac and in the pelvis. The complete blood picture and liver function tests were normal. The serum amylase (596 IU l^−1^) and serum lipase (396 IU l^−1^) values were found to be elevated.

**Figure 1.**
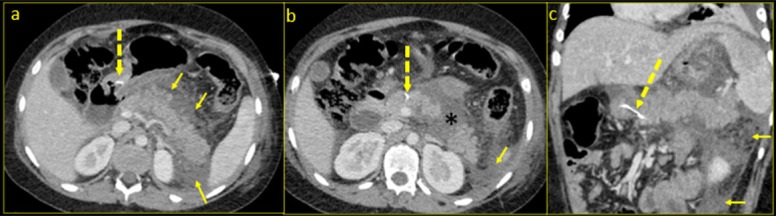
Axial CECT at the level of body of pancreas (**a**), at the level of head and body caudal to section 1a (b) and coronal-curved MPR images (**c**). The pancreas is bulky and oedematous with fat stranding and fluid in the lesser sac, left pararenal and lateral conal spaces (small yellow arrows). A focal area of necrosis in the form of parenchymal non-enhancement (black Asterix in b) is seen in the body of pancreas. The dashed yellow arrow in Figure 1a, b and c points to a curvilinear high density foreign body, whose proximal end is in the antropyloric canal of stomach (**a**) and distal end in embedded in the head of pancreas (b). CECT, contrast-enhanced CT; MPR, multiplanar reconstruction.

Contrast-enhanced CT scan of the abdomen revealed a bulky and oedematous pancreas with a 4 × 3.5 cm area of parenchymal necrosis in the distal body (Figure 1). There was fat stranding and free fluid in the lesser sac and in the left anterior pararenal space, caudally extending along the left lateral conal and Gerota’s fascia with free fluid in the pelvis (Figure 1). A thin curvilinear high density foreign body of 3.5 cm length was noted in the antral region of stomach (Figure 1), extending across the posterior wall and piercing into the pancreatic parenchyma in the region of head, cranial to the level of the pancreatic duct. There was no pneumoperitoneum. A diagnosis of acute necrotising pancreatitis caused by transgastric migration of a penetrating foreign body was made. The child denied conscious intake of any foreign body and so did the family.

## Treatment and outcome

**Figure 2.**
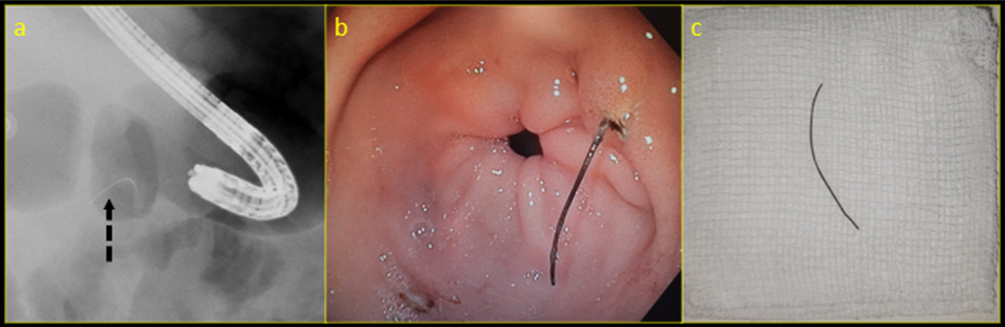
(a) Fluoroscopy of the upper abdomen in left lateral position (with endoscope in the stomach) demonstrates a thin curvilinear high-density object at the level of antropyloric canal (dashed black arrow). (b) Endoscopy demonstrated a thin metal wire in the gastric antrum perforating the posterior wall. (c) A 3.5 cm curvilinear metal wire was extracted with a rat tooth forceps.

A fluoroscopic examination was performed by the Department of Medical Gastroenterology, which showed a thin curvilinear high density foreign body in the upper abdomen corresponding to the level of gastric antrum (Figure 2a). Endoscopy showed a sharp curvilinear metallic wire piercing the posterior wall of antrum of the stomach (Figure 2b). This was removed intact as a single piece with rat tooth biopsy forceps (Figure 2c). He was then conservatively managed for pancreatitis, improved symptomatically, and was discharged in stable condition after 5 days.

Informed consent for the case to be published (including case history, data and images) was obtained from the child’s father.

## Discussion

Of the various causes of pancreatitis, trauma by blunt or penetrating injury is an uncommon cause. Even more unusual and infrequent is direct traumatic injury from an ingested foreign body perforating the upper GI tract.^1,2^ ^3^

While a majority of the ingested foreign bodies pass in the faeces, only about 1% are known to cause perforation.^1^ ^2^ Apart from the cricopharyngeal junction and the oesophageal sphincter, points of angulation or narrowing in the upper gastrointestinal (GI) tract such as the pylorus, duodenal curve and the ligament of Treitz are the common sites of perforation.^4^ The perforation may either be acute or chronic, at times with the foreign body getting embedded and taking months to erode through the gastric or duodenal wall, hence the timeline of presentations of symptoms can widely vary.^5^ There are very rare instances of these foreign bodies migrating to the pancreas; these may be asymptomatic and get incidentally detected on imaging or present with pain abdomen, pancreatitis, complications such as pseudoaneurysm, abscess and pseudotumour.^1,2,6–8^ One of the earliest literature of ingested foreign bodies perforating the upper GI tract and migrating into the pancreas was by Hashmonai et al, who reported a series of 10 cases of upper GI perforation by sewing needle, out of which four had perforated the duodenum and migrated into the pancreas.^9^

On review of literature, we found 20 case reports of pancreatic involvement by ingested sharp objects such as fishbone, sewing needle and toothpick penetrating the stomach or duodenum, out of which six patients had developed features of pancreatitis. Table 1 summarizes these case reports. However, we found only three case reports of ingested metallic wires perforating the upper GI tract and penetrating the pancreas, out of which one patient had developed signs of pancreatitis. Wu et al. in 2006 reported a similar case of a 45/M with pain abdomen and with rising amylase and lipase levels, in whom a piece of wire had perforated the stomach and migrated into the head of the pancreas, resulting in a peripancreatic abscess. The wire was detected on CT and removed on laparoscopy and there was no known history of its ingestion by the patient.^4^ Hao et al. in 2022 reported the case of 36/F presenting with dull epigastric pain, whose CT demonstrated a similar liner high density foreign body extending from the posterior gastric wall and embedded in the pancreas; a 3 cm metallic wire was removed on surgery and was assumed to have been ingested from remnants of a metallic brush used to clean pots and pans.^25^ There was, however, no abscess formation/ inflammation or pancreatitis in this patient. Sulieman et al. in 2022 reported a case of an accidentally ingested metallic grill brush wire masquerading as peripancreatic and paraduodenal inflammation on CT. Since the serum amylase and lipase values were normal, an MRI of the upper abdomen was performed for further evaluation, which revealed a metallic object presenting as susceptibility artefact within the paraduodenal inflammation; on push enteroscopy, a metallic brush wire/bristle was found perforating the third part of duodenum and extracted.^7^

**Table 1.** List of case reports in chronological order on ingested fishbones, sewing needles and toothpicks that perforated the upper GI tract and involved the pancreas

Serial number	Author	Year	Number of patients	Nature of foreign body	Site of perforation and migration	Clinical or imaging findings of pancreatitis (present/ absent)
1	Hashmonai et al.^9^	1978	4	Sewing needles	Duodenal perforation with extension to head of pancreas	Absent
2	Cheah et al.^10^	1999	1	Fish bone	Gastric wall perforation, embedded in head of pancreas	Not available
3	Pezzilli et al.^11^	2000	1	Sewing needle	Duodenal perforation with extension to head of pancreas	Absent
4	Toyonaga et al.^12^	2001	1	Sewing needle	Duodenal perforation with extension to head of pancreas	Absent
5	Rahalkar et al.^13^	2003	3	Sewing needle	Gastric wall perforation piercing body of pancreas	Not available
6	Kim et al.^3^	2004	1	Toothpick	Duodenal perforation, migrating to pancreas, causing splenic artery pseudoaneurysm	Present
7	Goh et al.^14^	2004	1	Fishbone	Gastric wall perforation, piercing the body of pancreas with a local abscesses mimicking malignancy	Present
8	Wang et al.^8^	2008	1	Fishbone	Abscess in pancreatic body mimicking a mass, possibly from gastric perforation	Absent
9	Yasuda et al.^15^	2010	1	Fish bone	Duodenal perforation with extension to head of pancreas	Absent
10	Yadav et al.^16^	2013	1	Sewing needle	No detectable bowel perforation, needle in head of pancreas	Absent
11	Symeonidis et al.^17^	2012	1	Fish bone	Duodenal perforation with extension to head of pancreas	Present
12	Shan Hu et al.^18^	2012	1	Fishbone	Gastric antral perforation, penetrating neck of pancreas	Present
13	Huang et al.^19^	2013	1	Fish bone	Peripancreatic collection containing fish bone. Site of perforation not mentioned	Absent
14	Gharib et al.^20^	2015	1	Fishbone	Duodenal perforation with extension to pancreas causing focal pancreatitis and SMV thrombosis	Present
15	Sae Byeol Choi et al.^21^	2016	1	Toothpick	Duodenal perforation with inflammatory pseudomass in head of pancreas	Absent
16	Mima et al.^22^	2018	1	Fishbone	Gastric antral perforation, embedded in pancreas	Absent
17	Jain et al.^1^	2018	1	Sewing needle	No detectable bowel perforation, needle in head of pancreas	Absent
18	Dal et al.^23^	2018	1	Sewing needle	Gastric perforation with extension to head of pancreas	Absent
19	Wang et al.^24^	2021	1	Fishbone	Gastric perforation and migrating into the neck of the pancreas	Absent
20	Yu et al.^6^	2022	1	Toothpick	Gastric perforation and migrating into the pancreas	Present

GI, gastrointestinal; SMV, superior mesenteric vein.

An accidentally or unknowingly ingested foreign body perforating the GI tract may at times be asymptomatic or present with vague symptoms several years later, but the potential morbidity resulting from a migrating foreign body penetrating the pancreas can be significant.^2,3,5^

Imaging with CT of the abdomen plays a major role in diagnosis. The presence of a small linear high-density object located intramurally, outside the bowel or embedded in the viscera in any patient with upper abdominal pain should raise the suspicion of perforated foreign body from upper GI tract, even in the absence of a history of foreign body ingestion at the time of presentation. The presence of such an object within a collection or phlegmonous mass is even more characteristic. Imaging can not only aid in the diagnosis but also in the decision-making between endoscopic extraction *vs* laparoscopy or laparotomy for extraction.^1^ The proximal end of the curvilinear foreign body was seen withing the gastric lumen on CT in our case, thus helping expedite the process of endoscopic retrieval. Indirect signs such as presence of surrounding fluid collection, air pockets, peritoneal inflammation in the vicinity or bowel wall oedema can point to the site of perforation even when the foreign body has migrated.^5^

Though the imaging findings were characteristic of a foreign body on CT in our case and its presence and nature were demonstrated on endoscopy and upon its extraction, there was no history of its ingestion.

Poorly conspicuous small calibre foreign bodies may pose a great diagnostic challenge, as the surrounding inflammation may obscure its visibility on CT. In such cases, further evaluation with MRI may help detect the presence of foreign body through the susceptibility artefact.^7^

## Learning points

1. This case showcases a very unusual cause of pancreatitis form traumatic injury caused by a metal wire perforating through the stomach and piercing the pancreas.
2. CT scan of the abdomen plays a vital role in the diagnosis of foreign body causing bowel perforation by demonstrating a small linear or curvilinear high-density object with or without surrounding collection, fat stranding and oedema in the adjacent bowel wall. The exact location of the foreign body, presence of any intraluminal component within the GI tract, presence of surrounding fat-stranding, collection or a phlegmonous mass must be included in the report to aid in planning the treatment.
3. History of foreign body ingestion at the time of clinical presentation/imaging may not always be present; absence of such history in the presence of highly suggestive radiological findings must not stop the radiologist from making a diagnosis of perforated foreign body for the following reasons: (a) The patients are likely to ingest tiny metallic wires/needles unknowingly. (b) Patients can be asymptomatic despite a perforation. (c) Chronic perforations can present late, at times after months to years from the time of ingestion.

## References

[1] Jain A, Nag HH, Goel N, Gupta N, Agarwal AK . Laparoscopic removal of a needle from the Pancreas. J Minim Access Surg 2013; 9: 80–81. doi: 10.4103/0972-9941.110968 23741114 PMC3673579

[2] Williams HE, Khokhar AA, Rizvi M, Gould S . Gastric Perforation by a foreign body presenting as a Pancreatic Pseudotumour. Int J Surg Case Rep 2014; 5: 437–39. doi: 10.1016/j.ijscr.2014.04.021 24926924 PMC4064400

[3] Kim KH, Woo EY, Rosato EF, Kochman ML . Pancreatic foreign body: ingested toothpick as a cause of Pancreatitis and hemorrhage. Gastrointest Endosc 2004; 59: 147–50. doi: 10.1016/s0016-5107(03)02364-2 14722574

[4] Wu C, Hungness ES . Laparoscopic removal of a Pancreatic foreign body. JSLS 2006; 10: 541–43. 17575779 PMC3015777

[5] Kuzmich S, Burke CJ, Harvey CJ, Kuzmich T, Andrews J, Reading N, et al . Perforation of gastrointestinal tract by poorly conspicuous ingested foreign bodies: radiological diagnosis. Br J Radiol 2015; 88: 20150086. doi: 10.1259/bjr.20150086 25827210 PMC4628459

[6] Yu S, Su S, Shao X, Zhou Y, Yu Y, Kuai X, et al . Misdiagnosis of acute Pancreatitis in a patient with foreign body ingestion: a case report and literature review. Am J Transl Res 2022; 14: 8286–91. 36505331 PMC9730075

[7] Sulieman M, Hall MAK, Wong G, Ahmed R . When it's not Pancreatitis, don't brush it off: A case report of small bowel Perforation caused by a grill brush bristle masquerading as Pancreatitis. Cureus 2022; 14: e30422. doi: 10.7759/cureus.30422 36407171 PMC9670664

[8] Wang WL, Liu KL, Wang HP . Clinical challenges and images in GI. Pancreatic abscess resulting from a fish bone penetration of the stomach. Gastroenterology 2008; 135: 1865–2160. doi: 10.1053/j.gastro.2008.10.067 19007782

[9] Hashmonai M, Kaufman T, Schramek A . Silent perforations of the stomach and duodenum by needles. Arch Surg 1978; 113: 1406–9. doi: 10.1001/archsurg.1978.01370240028004 736772

[10] Cheah WK, Mar Fan MJ, Goh PM . Laparoscopic removal of fish bone. Surg Laparosc Endosc Percutan Tech 1999; 9: 223–25. 10804007

[11] Pezzilli R, Barakat B, Re G, Bertaccini P . Foreign body of the Pancreas. Dig Liver Dis 2000; 32: 179. doi: 10.1016/s1590-8658(00)80407-7 10975795

[12] Toyonaga T, Shinohara M, Miyatake E, Ouchida K, Shirota T, Ogawa T, et al . Penetration of the duodenum by an ingested needle with migration to the Pancreas: report of a case. Surg Today 2001; 31: 68–71. doi: 10.1007/s005950170224 11213048

[13] Rahalkar MD, Pai B, Kukade G, Al Busaidi SS . Sewing needles as foreign bodies in the liver and Pancreas. Clin Radiol 2003; 58: 84–86. doi: 10.1053/crad.2002.1118 12565211

[14] Goh BKP, Jeyaraj P-R, Chan H-S, Ong H-S, Agasthian T, Chang KTE, et al . A case of fish bone Perforation of the stomach mimicking a locally advanced Pancreatic carcinoma. Dig Dis Sci 2004; 49: 1935–37. doi: 10.1007/s10620-004-9595-y 15628728

[15] Yasuda T, Kawamura S, Shimada E, Okumura S . Fish bone penetration of the duodenum extending into the Pancreas: report of a case. Surg Today 2010; 40: 676–78. doi: 10.1007/s00595-009-4110-x 20582523

[16] Yadav TD, Singh H, Sah R . Unusual foreign body of Pancreas: surgical management. JOP 2013; 14: 669–70. doi: 10.6092/1590-8577/1902 24216558

[17] Symeonidis D, Koukoulis G, Baloyiannis I, Rizos A, Mamaloudis I, Tepetes K . Ingested fish bone: an unusual mechanism of Duodenal Perforation and Pancreatic trauma. Case Rep Gastrointest Med 2012; 2012: 308510. doi: 10.1155/2012/308510 22919520 PMC3420078

[18] Shan Hu DH . A gastrointestinal foreign body induced Pancreatitis. [CASE 9747]. Eurorad 2012.

[19] Huang YH, Siao FY, Yen HH . Pre-operative diagnosis of Pancreatic abscess from a penetrating fish bone. QJM 2013; 106: 955–56. doi: 10.1093/qjmed/hcs166 22927535

[20] Gharib SD, Berger DL, Choy G, Huck AE . CASE RECORDS of the MASSACHUSETTS GENERAL HOSPITAL. case 21-2015. A 37-year-old American man living in Vietnam, with fever and bacteremia. N Engl J Med 2015; 373: 174–83. doi: 10.1056/NEJMcpc1411439 26154791

[21] Sae Byeol Choi HJL, Choi SY . Intraabdominal abscess due to Duodenal Perforation by a toothpick, mimicking a Pancreatic cancer. Journal of the Pancreas 2016; 17: 549–52.

[22] Mima K, Sugihara H, Kato R, Matsumoto C, Nomoto D, Shigaki H, et al . Laparoscopic removal of an ingested fish bone that penetrated the stomach and was embedded in the Pancreas: a case report. Surg Case Rep 2018; 4(): 149. doi: 10.1186/s40792-018-0559-4 30594971 PMC6311171

[23] Dal F, Hatipoğlu E, Teksöz S, Ertem M . Foreign body: A sewing needle migrating from the gastrointestinal tract to Pancreas. Turk J Surg 2018; 34: 256–58. doi: 10.5152/turkjsurg.2017.3391 30302435 PMC6173595

[24] Wang Y, Luo X, Zhang J . Successful Laparoscopic treatment for sustained abdominal pain due to fish bone migrating into the neck of the Pancreas: a case report and thinking about surgical approach through the literature review. Surg Case Rep 2021; 7(): 91. doi: 10.1186/s40792-021-01174-y 33851276 PMC8044272

[25] Hao F, Feng Q, Li J, Wu H . An ingested metallic wire migrating from stomach to Pancreas treated by Laparoscopic surgery: A case report. Front Surg 2022; 9: 927637. doi: 10.3389/fsurg.2022.927637 36684257 PMC9852039

